# Empirical Equation Based Chirality (n, m) Assignment of Semiconducting Single Wall Carbon Nanotubes from Resonant Raman Scattering Data 

**DOI:** 10.3390/nano3010001

**Published:** 2012-12-24

**Authors:** Md Shamsul Arefin

**Affiliations:** Department of Electrical Engineering and Computer Science, Northern Arizona University, South San Francisco Street, Flagstaff, AZ 86011, USA; E-Mail: msa59@nau.edu; Tel.: +1-928-814-5931;Fax: +1-928-523-2300.

**Keywords:** chiral index, chirality assignment, single wall carbon nanotube, resonant Raman spectroscopy, optical transition energy, tight-binding model, nearest-neighbor hopping parameter

## Abstract

This work presents a technique for the chirality (*n*, *m*) assignment of semiconducting single wall carbon nanotubes by solving a set of empirical equations of the tight binding model parameters. The empirical equations of the nearest neighbor hopping parameters, relating the term (2*n*− *m*) with the first and second optical transition energies of the semiconducting single wall carbon nanotubes, are also proposed. They provide almost the same level of accuracy for lower and higher diameter nanotubes. An algorithm is presented to determine the chiral index (*n*, *m*) of any unknown semiconducting tube by solving these empirical equations using values of radial breathing mode frequency and the first or second optical transition energy from resonant Raman spectroscopy. In this paper, the chirality of 55 semiconducting nanotubes is assigned using the first and second optical transition energies. Unlike the existing methods of chirality assignment, this technique does not require graphical comparison or pattern recognition between existing experimental and theoretical Kataura plot.

## 1. Introduction

Semiconducting single wall carbon nanotubes (SWCNTs) already emerged as a promising candidate for photovoltaic applications [1,2,3,4,5,6], opto-electronics [7,8], and molecular electronics [9,10,11]. A number of advanced applications such as transistor memory [9], field-effect transistors [10], and near-infrared optical sensors [11] require nanotube samples with little or no structural variation. A SWCNT (*n*, *m*) is metallic if *mod*(*n*− *m*, 3) = 0 and semiconducting if *mod*(*n*− *m*, 3) =1 or 2 (commonly termed as mod1 or mod 2 type, respectively) [12]. This relation is always found true except for SWCNT with a very small diameter, where curvature effect dominates its properties [13]. Since the electronic and the optical properties of SWCNT vary greatly with their chirality, the experimental determination of the chirality (*n*, *m*) of SWCNT has been a challenge ever since their discovery [14,15]. Identification of spectroscopic features and correlating them with nanotube geometric structure is always necessary to separate, sort, and identify nanotubes after their production [12,16]. Methods including dielectrophoresis [17,18], centrifugation [19,20,21], chromatography [22,23,24], selective solubilization [25,26], and selective reaction [27,28] are devised for the separation and sorting of semiconducting SWCNTs from metallic nanotubes. After the separation, the immediate next challenge is to identify the chiral index (*n*, *m*) of each semiconducting SWCNT. As the fabrication techniques are not yet in the position to deliver nanotubes of specific chirality, there is still a need for reliable techniques for the identification of the chirality of a given nanotube. 

Since the diameter of an individual SWCNT is determined by its chirality, (*n*, *m*), there have been a number of experimental approaches based on TEM [29], STM [30,31], or the radial breathing mode (RBM) in Raman spectroscopy [15,16] to determine the diameter of a given nanotube. In principle, an exact knowledge of the diameter allows the determination of the chiral indices of a tube. However, the diameters area multi-valued function of chirality (*n*, *m*) and are too closely spaced for such a procedure to work. Moreover, as two different SWCNTs may have the same diameter, the unique assignment of chirality only from the diameter is impossible. Hence, at least one more piece of information is required for unique assignment of the chirality of nanotube [15]. 

Resonant Raman scattering (RRS) [15,16,32,33,34,35,36,37,38,39,40,41,42,43], Rayleigh scattering [44,45,46,47,48], and photoluminescence (PL) excitation [49,50,51,52,53] have been the mainstream tools for non-destructive chirality characterization, and each method has unique capabilities. Each method uses at least two pieces of information for the unique assignment of chirality [37,41,42]. First, RRS method uses one optical transition energy, (*E_ii_*) and the nanotube RBM frequency, *ω_rbm_*. Second, Rayleigh scattering uses electron diffraction. Lastly, PL method uses optical absorption and emission energies for unique chirality assignment [37]. Bachilo *et al.* [50] showed PL based effective chirality (*n*, *m*) assignment based on pattern recognition between experimental and theoretical (derived from the extended tight binding model) plot of the second transition (excitation) energy versus the first transition (emission) energy. It can be noted that all these methods commonly use the information about one of the transition energies. Though the spectroscopic experiments are different in these methods, they bear some similarities in principle. All of them follow a laborious mapping of the observed properties of the produced batch of SWCNTs with an existing theoretical plot that depicts the same properties to find one-to-one correspondence of chirality. Therefore, the methods need to go through some kind of pattern recognition process and need a prior graphical plot to make final assignment. Moreover, pattern recognition is possible simply if the Raman spectrum shows a set of different RBMs. This is merely the case for samples containing different kinds of nanotubes that are produced as ensembles of nanotubes. There is no scope for pattern recognition, if all observed Raman spectra shows just one RBM for an isolated tube or if the sample contains only one kind of chiral indices (*n*, *m*). In such case, quality of the assignment fully depends on the chosen theoretical plot and may lead to ambiguity. 

RRS is quite reliable, straightforward (though laborious and expensive), and hence most widely used for chiral index assignment. Moreover, RRS can be performed in resonance with the second optical transition, which is in the visible energy range. Thus, no infrared-sensitive spectrometers and detectors are needed [37]. RRS provides the information of the optical transition energies (*E_ii_*) and the RBM frequency (*ω_rbm_*). The RBM frequency gives the diameter (*d_t_*) as they are inversely related through a semi-empirical equation [16,33,34,50,54,55]. Since the (*E_ii_*, *ω_rbm_*) pair is unique for each SWCNT, proper structural assignment can be made using a prior theoretical model for (*E_ii_*, *ω_rbm_*) to (*n*, *m*) transformation [42]. Earlier approach was to plot all *E_ii_* versus *d_t_* (from *ω_rbm_*) to form an experimental Kataura plot that is mapped with an existing theoretical Kataura plot to give one-to-one correspondence for each chirality [32,33]. Unfortunately, the uniqueness of this transformation may be hampered in this process by the possible error involved in the empirical calculation of *d_t_* from *ω_rbm_*. Hence, alternative methods were proposed later by many authors where all *E_ii_* are plotted directly against corresponding *ω_rbm_* [42,43] or inverse *ω_rbm_* [15,36,37] (instead of *d_t_*) and compared with a theoretical plot of (*E_ii_*,*ω_rbm_*) or (*E_ii_*, 1/*ω_rbm_*). Then pattern recognition between two plots is performed by stretching and shifting the vertical and horizontal axes of the plots until good correspondence between the experimental points and the theoretically calculated values is achieved [15,36,37]. By this process, assumption of empirical parameters for calculating *d_t_* from *ω_rbm_* can be avoided and possible theoretical or experimental error can be neutralized within a limit. Though this pattern recognition finally gives a unique one-to-one correspondence for each chiral index (*n*, *m*), it requires to follow the pattern recognition approach and also needs a prior graphical plot or tabulated data. 

This paper presents an empirical equation based novel technique for SWCNT chirality assignment where the chiral index (*n*, *m*) can be directly found by solving the empirical equations of the nearest neighbor hopping parameters. So far, no such equation based technique is proposed for chirality assignment. Like other methods, it also requires the information of optical transition energy and nanotube diameter (*d_t_*). However, unlike other methods, it does not follow graphical approach and does not need a prior tabulated data or graphical plot. This method is applicable for any semiconducting SWCNT, whether isolated or in a batch. 

The remainder of this article is organized as follows. A set of Empirical equations of the nearest neighbor hopping parameters for calculating the first and second optical transition energies are formulated in Section 2. Section 3 describes the proposed empirical equation based chirality assignment technique using both the first optical transition energy and the second optical transition energy. The empirical equations are used from reverse direction for chirality assignment. An algorithm to determine the chiral index (*n*, *m*) of unknown SWCNT from RRS experiment is also discussed. The supporting empirical equations for calculating chiral indexes are presented in Appendix A and Appendix B. The numerical results for chirality assignment of 28 semiconducting tubes using the first optical transition energy and the RBM frequency values and 27 semiconducting tubes using the second optical transition energy and the RBM frequency values are presented in Section 4. Finally, Section 5 presents the conclusions. 

## 2. ModifiedTight Binding Model

In this section, a set of empirical equations are formulated that can predict the first (*E*_11_) and second (*E*_22_) optical transition energies accurately. This section serves as the first step of empirical equation based chirality assignment. In the next section, the empirical equations that are used from reversed direction for chirality assignment are presented. 

The simple expression [45] of optical transition energies from Tight Binding model with the nearest-neighbor approximation is

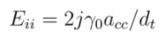
(1)
where γ_0_ is the nearest-neighbor hopping parameter, *a_cc_* = 0.144 nm is carbon-carbon bond length, *d_t_* is nanotube diameter in nm, given by *d_t_* = 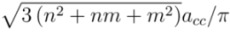, and *n* is an integer. This equation gives first optical transition energy for *i* = 1, *j* =1, 


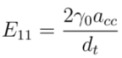
(2)

Since the experimental observation showed that the ratio of *E*_22_ to *E*_11_ deviates from 2, the ratio is termed as “*r*” [56]. Putting *i* =2, *j* = *r* in Equation (1) gives the expression of second optical transition energy (*E*_22_) as,


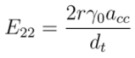
(3)

Equation (1) shows good qualitative agreement with experimental results but fails quantitatively [57,58,59]. This is because, it originates from a too simple model of SWCNT that ignores important experimental observations like “curvature effect”, “chirality effect”, “trigonal warping effect”, and “many body effect” (electron-electron interaction) [60,61,62,63,64]. The first factor, “Curvature effect”, is induced by *σ*-π re-hybridization and de-localized π-bond orbitals and corresponding band structure deviation from simple π-orbital graphene picture [13,57,59,60,61]. It causes quantitative difference and also turns some *mod*(*n*− *m*, 3) = 0 tubes into quasi-metallic or small bandgap semiconductors [59]. The effect is stronger for small diameter tubes due to their bigger curvature [59]. Since diameter *d_t_* is fully responsible for tube curvature, suitable inclusion of *d_t_* term can reﬂect this effect. The second factor is “chirality effect” that originates from individual nanotube chirality and responsible for some unique features of each tube. As classifying semiconducting SWCNT in mod1 and mod2type originates from chirality (*n*, *m*), not from chiral angle, chirality effect can be reﬂected through alternative combination of chiral index. The third factor is “trigonal warping effect” [64,65] that arises from both curvature and chirality and causes difference in transition energies for mod 1 and mod 2 types. It also depends on *d_t_*. Hence, the *d_t_* term in the empirical equation can account the effects. The fourth factor is “many body effect” (electron-electron interaction) or “self-energy and exitonic effect” [28] that causes “ratio problem” and “blue shift problem”. This effect is adjusted within numerical fitting parameters. Since the nature and amount of these effects are still being discussed in literatures and also much disputed, any pre-defined or specific term cannot be included to explain these effects. E_11_ and E_22_ calculated from Equations (2) and (3) deviates up to 25% from experimentally observed value if γ_0_ is considered as constant [40]. Another experimental observation is, with comparable diameter, transition energies for mod 1 type semiconducting SWCNTs are smaller than mod 2 type for odd transitions, but higher for even transitions [65], *i.e*., E_11_*^mod^*^1 ^< E_11_*^mod^*^2^ but E_22_*^mod^*^1 ^< E_22_*^mod^*^2^. Equations (2) and (3) cannot reﬂect this observation also. Many authors tried to improve TB model theoretically to give better prediction of experimental observations. Some of them proposed to add extra terms with Equations (2) and (3) to reﬂect curvature effect in terms of *d_t_* and chirality effects in terms of chiral angle, *θ* [15,48,61,62]. By including cos(3*θ*) term, these nonlinear scaling relations showed good match between experimental and calculated optical transition energies [48,66]. However, one common factor to express chirality effect in all the above-mentioned theoretical and empirical equations is a specific term cos(3*θ*) only [15,38,62] and did not consider any other combination of chiral indices. Therefore, the objective of the proposed equations is to express the optical transition energies in terms of *d_t_* and (*n*, *m*). 

The overall issues are addressed empirically by including “curvature effect”, “chirality effect”, “trigonal warping effect”, and “many body effect” in γ_0_, instead of directly adding those with TB model, such that the basic form of TB model remains intact. Therefore, Equations (2) and (3) may be improved by taking γ_0_ as a curvature and chirality dependent parameter, *i.e*., including all these observed effects in γ_0_ so as to predict experimental values accurately. After following experimental values of *E*_11_ and *E*_22_ for a large number of semiconducting SWCNTs [32,33,34,35,36,37,38,39,40,41,42,43], it is found that they are very sensitive to a specific chiral index combination (2*n*− *m*). Based on this observation and taking some insights from equations proposed by other authors [37,40,50,61,64] to improve Equations (2) and (3), the following set of empirical equations for γ_0_ and (rγ_0_) is formulated to predict *E*_11 _and *E*_22_ of semiconducting SWCNTs with higher accuracy. 

To calculate *E*_11_ for mod 1 type:

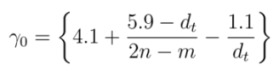
(4)


To calculate E_11_ for mod 2 type :

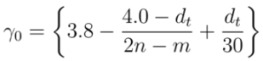
(5)


To calculate *E*_22_ for mod 1 type :

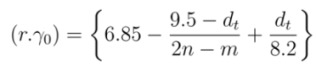
(6)


To calculate *E*_22_ for mod 2 type :

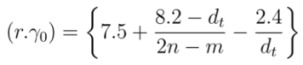
(7)


In these empirical expressions [Equations(4)-(7)] of γ_0_ and *r*γ_0_,“curvature effect”, “chirality effect”, “trigonal warping effect”, and “self-energy and exitonic effect” are included by arranging nanotube diameter, *d_t_*, and a term, (2*n*− *m*), with numerical fitting parameters. Separate equations are proposed for mod 1 and mod 2 types to reﬂect their relative difference in transition energies and also to trace their unique trends. 

**Figure 1:**
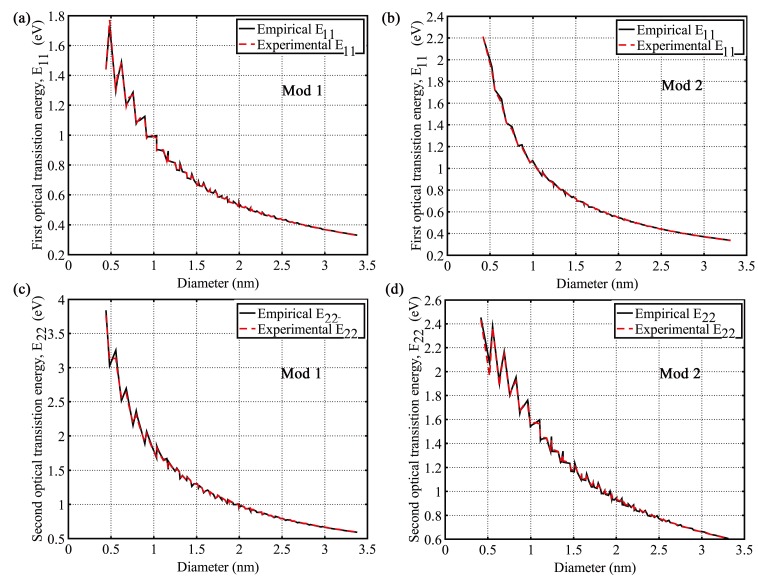
Plot of first (*E*_11_) and second (*E*_22_) optical transition energy *vs*. nanotube diameter (*d_t_*). (**a**) Comparing experimental and empirical *E*_11_ for mod 1; (**b**) Comparing experimental and empirical *E*_11_ for mod 2; (**c**) Comparing experimental and empirical *E*_22_ for mod 1; (**d**) Comparing experimental and empirical *E*_22_ for mod 2.

**Figure 2:**
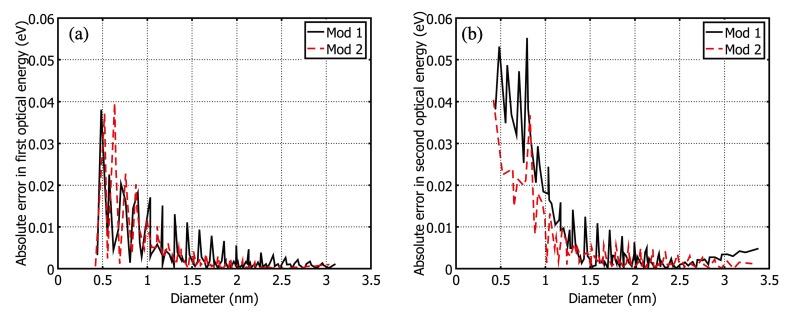
Plot of absolute deviations (errors) of first (*E*_11_) and second (*E*_22_) optical transition energy *vs*. nanotube diameter (*d_t_*). (**a**) Absolute deviations of empirical *E*_11_ from experimental data for both mod 1 and mod 2; (**b**) Absolute deviations of empirical *E*_22_ from experimental data for both mod 1 and mod 2.

All the semiconducting SWCNTs that fall within theoretically possible minimum and maximum diameter range were considered. Values of the first and second optical transition energies (eV) corresponding to all these semiconducting SWCNTs were recorded from multiple reports of different optical spectroscopic experiments [14,16,33,34,35,36,37,38,39,41,42,43,44,45,50,65]. First and second optical transition energies (*E*_11_ and *E*_22_) of all 212 semiconducting SWCNTs for mod 1 and mod 2 types were calculated from Equations (2),(4), and (5) and Equations (3), (6), and (7), respectively. The calculated *E*_11_ and *E*_22_ showed excellent match from lowest diameter (0.4nm) to highest diameter (3nm). The plots of *E*_11_ *vs*. *d_t_* and *E*_22_ *vs*. *d_t_* for both mod 1 and mod 2 types are shown in Figure 1. The calculated values of *E*_11_ and *E*_22_ from Equations (2) and (3) provides the almost same level of accuracy with experimental results over the full diameter range. The absolute deviations (errors) of empirical data of *E*_11_ and *E*_22_ from experimental data that reduce more for increasing diameters, are shown in Figure 2.The agreement between experimental and empirical graphs over the full diameter range is so good as if they are replica of each other. 

The overall comparison between the empirical data and experimental data is summarized in Table 1 and Table 2 for *E*_11_ and *E*_22_, respectively. Table 1 shows that average absolute deviations (| ∆E |) and % average absolute deviations (% | ∆E |) of empirical data for * E*_11_ are very low and within tolerable margin. Average error for *E*_11_ over full diameter range is only 0.0036 eV ( 0.43%) and 0.0033 eV (0.32%) for mod 1 and mod 2, respectively. Same things can be noticed from Table 2, where average error for *E*_22_ over full diameter range is only 0.0113eV (0.65%) and 0.0081 eV (0.56%) for mod 1 and mod 2, respectively. In both cases, | ∆E | and % | ∆E | reduces more for increasing diameters as shown in Table 1 and Table 2. 

**Table 1:** Comparison of experimental and empirical data of *E*_11_ and corresponding average error and % average error.

	MOD 1 Type		MOD 2 Type	
**Diameter, ** *d_t _* **(nm)**	**Average **	**Average **	**Average **	**Average **
	| Δ *E* |	% | Δ *E* |	| Δ *E* |	% | Δ *E *|
0.4 ≤ *d_t_* ≤ 3.0	0.0036	0.43%	0.0033	0.32%
1.0 ≤ *d_t_* ≤ 3.0	0.0023	0.36%	0.0015	0.20%
1.5 ≤ *d_t_* ≤ 3.0	0.0015	0.29%	0.0006	0.11%

The empirical expressions of γ_0_ and rγ_0_ enable Equations (2) and (3) to give almost accurate prediction of the first and second optical transition energies of semiconducting SWCNTs and remove its various shortcomings. Though nonlinear scaling relations that include *cos*(3*θ*) term provide almost same level of accuracy with this proposed empirical equations, the advantage of these equations is that it can reﬂect the chirality effect through chiral index (*n*, *m*) instead of chiral angle *θ*. Moreover, it gives almost same level of accuracy for lower and higher diameters and hence, strengthens the nearest neighbor tight binding model that is commonly accused for being highly inaccurate in lower diameter tubes. Therefore, the approach of taking γ_0_ as a parameter whose value will depend on various observed effects is quite justified and will be proven more effective than similar earlier attempts. Moreover, the empirical equations can directly relate optical transition with nanotube chirality (*n*, *m*). 

**Table 2:** Comparison of experimental and empirical data of *E*_22_ and corresponding average error and % average error.

	MOD 1 Type		MOD 2 Type	
**Diameter, ** *d_t_* ** (nm)**	**Average**	**Average **	**Average**	**Average **
	| Δ*E *|	% | Δ*E *|	| Δ*E *|	% | Δ*E *|
0.4 ≤ *d_t_* ≤ 3.0	0.0115	0.66%	0.0083	0.57%
1.0 ≤ *d_t_* ≤ 3.0	0.0052	0.46%	0.0037	0.35%
1.5 ≤ *d_t_* ≤ 3.0	0.0037	0.39%	0.0031	0.33%

## 3. Method

The objective of proposing the empirical equations is to use these equations from reversed direction for the chirality assignment. Using experimental value of *E_ii_* (*E*_11_ or *E*_22_) and*d_t_ ,* the corresponding chiral index (*n*, *m*) of any unknown SWCNT can be determined. If *E*_11_ is available, Equations (2), (4), and (5) are used to find chiral index as discussed in Appendix A. The values of *n* from first optical transition energy for mod 1 and mod 2 are termed as 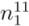 and 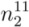 , respectively. From Equations (20) and (21), the values of 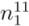 and 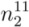 can be expressed as

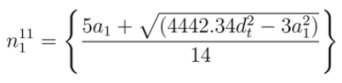
(8)

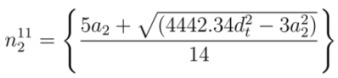
(9)
where *a*_1_ and *a*_2_ are found from Equations (18) and (19), respectively. In the same way, the values of *m *from first optical transition energy for mod 1 and mod 2 are termed as 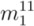 and 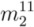 , respectively. From Equations (18) and (19), corresponding values of 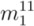 and 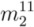 are

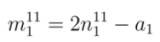
(10)

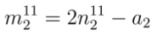
(11)


Equations (8)-(11) give two integer pairs, (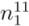, 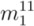) and (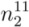, 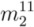), which are the candidates for chiral index of unknown SWCNT. 

In the same way, if *E*_22_ is available, Equations (3), (6), and (7) are used to find chiral index as discussed in Appendix B. The values of *n* from second optical transition energy for mod 1 and mod 2 are termed as 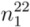 and 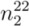, respectively. From Equations (24) and (25), the values of 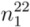 and 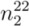 can be expressed as

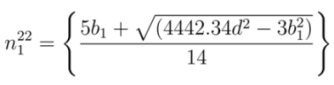
(12)

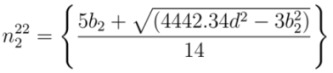
(13)
where *b*_1_ and *b*_2_ are found from Equations (22) and (23), respectively. Similarly, the values of *m* from second optical transition energy for mod 1 and mod 2 are termed as 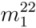 and 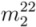 , respectively. From Equations (22) and (23), corresponding values of 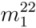 and 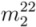 are

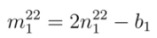
(14)

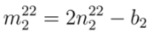
(15)
Note that the values of 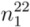 and 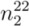 and corresponding 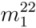 and 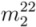 may become fractions. Finally, Equations (12)-(15) give two pairs (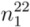, 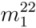) and (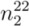, 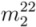) that are the candidates for chiral index of unknown SWCNT. 

**Figure 3:**
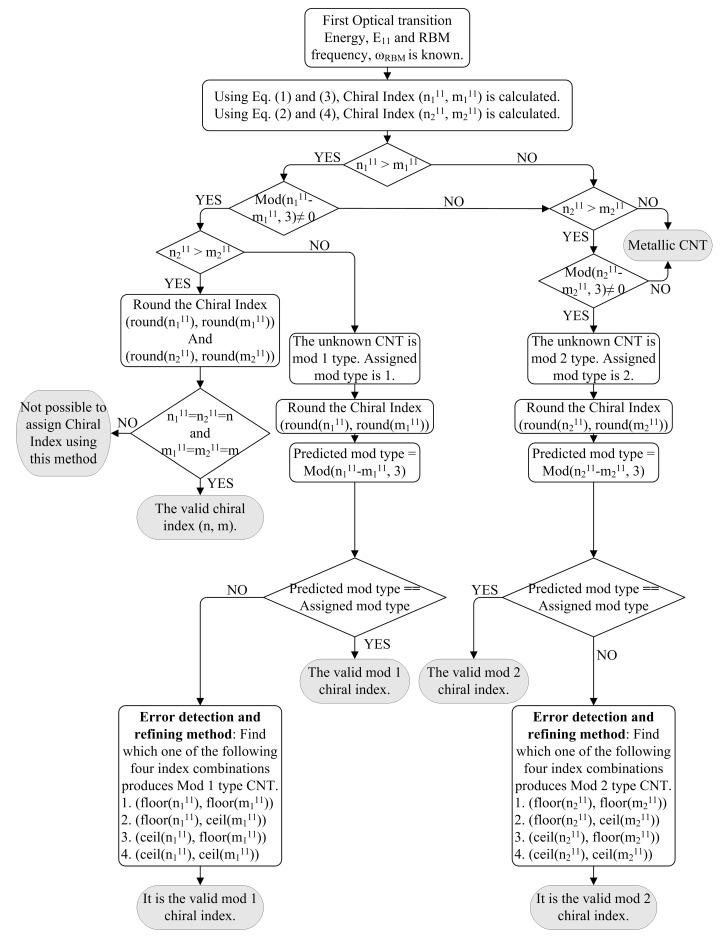
A ﬂow chart of the algorithm to determine the chiral index (*n*, *m*) of unknown SWCNT from RRS experiment (RBM frequency, *ω_rbm_*) for available first optical transition energy, *E*_11_. Same algorithm is used to determine chiral index for second optical transition energy, *E*_22_.

The required two pieces of information (*E*_11_ or *E*_22_ and *ω_rbm_*) will be taken from RRS experiments reported by numerous authors [15,16,32,33,34,35,36,37,38,39,40,41,42,43]. A ﬂow chart of the algorithm is shown in Figure 3 for finding the chiral index (*n*, *m*) from *E*_11_ and *ω_rbm_*. Assuming that *E*_11_ and *ω_rbm_* of the unknown SWCNT from RRS experiment are known, two integer pairs(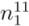, 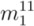) and (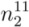, 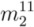) are calculated from Equations (8)-(11). The pair (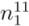 , 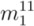) originates from mod 1 type’s equation, whereas the pair (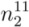 , 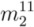) originates from mod 2 type’s equation. 

Since all the samples under test are semiconducting SWCNTs, they should always satisfy *n *> *m* and *mod*(*n*−*m*, 3) ≠ 0 conditions in the proposed algorithm. The ﬂow chart gives four distinct possibilities of values of these pairs. First, if 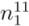 > 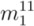 and 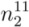 < 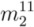, assigned mod type is mod 1. Rounding the pair (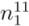, 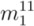), the predicted mod type is calculated using *mod*(*round*(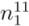) − *round*(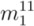), 3). If the assigned mod type is same as the predicted mod type, the rounded pair gives the actual mod 1 type chiral index. If the assigned mod type is not equal to the predicted mod type, error detection and refining method is applied on the (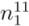, 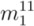) pair. Second, assigned mod type is mod 2 if 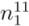 < 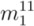 and 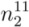 > 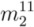. The predicted mod type is calculated using *mod*(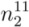
− 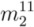, 3) after rounding the pair (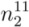 , 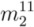).The rounded pair gives the actual mod 2 type chiral index if the assigned mod type is same as the predicted mod type. If the assigned mod type is not equal to the predicted mod type, error detection and refining method is applied on the (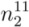, 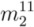) pair. Third, for 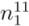 > 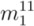 and 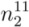 > 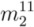 conditions, if rounding to nearest interger results *round*(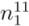) = *round*(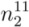) = *n* and *round*(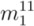) = *round*(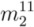) = *m*, the chirality of the unknown CNT is (*n*, *m*). Otherwise, the chirality of that unknown CNT cannot be determined. Finally, though the empirical equations are formulated for mod 1 and mod 2 SWCNT, it can be extended for metallic CNTs only to detect. If the unknown CNT is metallic rather than SWCNT, the values of (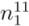, 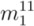) and (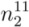, 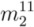) pairs follow the condition: 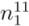 < 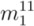 and 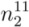 < 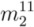. Since both conditions fail to satisfy the condition of semiconducting SWCNT, the sample is a metallic CNT. This condition is particularly useful to detect the presence of metallic CNT as clean separation of metallic CNT from semiconducting CNT is experimentally difficult. 

In the error detection and refining method stage of the algorithm, the pair is truncated and rounded to adjust small experimental or empirical error. Since the values of *n* and *m* are estimated by rounding, the values that have fraction parts of *n* and *m* within 0.40 to 0.60 are prone to wrong assignment. Such error may come from the error margin of our empirical equations or due to slight deviation in experimental data given by RRS. If the RRS data do not deviate enough, the valid pair is in the close vicinity of the actual chiral index. Therefore, rounding such values require some tie-breaking rule. The tie-breaking rule in this method is to round down (ﬂoor) and round up (ceil). For this purpose, error detection and refining method is adopted to verify each of the assigned chirality instantly by comparing predicted mod type and assigned mod type. The possible closest chiral index pairs for mod 1 are listed as four combinations of (*floor*(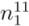), *floor*(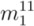)), (*floor*(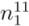), *ceil*(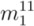)), (*ceil*(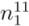), *floor*(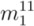)), and (*ceil*(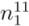 ), *ceil*(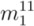 )). From this list, the one that matches with assigned mod type (mod 1) is reassigned as the true chiral index of that semiconducting SWCNT. Similarly, possible chiral index pairs for mod 2 are listed as four combinations and one pair is reassigned as true chiral index that matches with assigned mod type (mod 2). 

The same algorithm is followed for second optical transition energy. If *E*_22_ and *ω_rbm_* of the unknown SWCNT from RRS experiment are known, two pairs (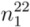, 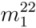) and (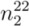, 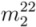) are calculated from Equations (12)-(15).

This method can assign the chirality of unknown semiconducting SWCNT by using only two variables: *E*_11_ and *ω_rbm_* or *E*_22_ and *ω_rbm_*. If all information is available (*E*_11_, *E*_22_, and *ω_rbm_*), this method will not provide simplified procedure for chirality assignment. Since the empirical equations for both *E*_11_ and *E*_22_ are presented here as a function of *d_t_* and (*n*, *m*), the ratio of optical transition energies (*E*_11_/* E*_22_) is also dependent on *d_t_* and (*n*, *m*). In this case, the procedure for chirality assignment will be same as the the proposed algorithm. However, it will be helpful in this case to determine and verify the chiral index using this algorithm, if the chirality assignment using one data cannot be determined. As stated earlier, if the conditions 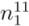 > 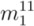 and 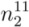 > 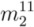 satisfy, but the conditions *round*(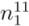) = *round*(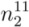) and *round*(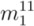) = *round*(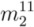) do not satisfy, the chirality of that CNT cannot be determined using this algorithm. In this situation, alternate optical transition energy information may determine the actual chirality. 

## 4. Results

The chirality of a total of 55 semiconducting tubes has been successfully assigned using this technique, among which 28 semiconducting tubes were assigned using *E*_11_ and *ω_rbm_* values and 27 semiconducting tubes were assigned using *E*_22_ and *ω_rbm_* values. All the prediction made using the current proposed method match with the CNT determination made in the published papers. Though some assignments did not match the assigned and predicted mod type initially, the error detecting and refining method detects and corrects those assignments. Finally, the proposed technique assigned the actual chiral index to all the tested samples. 

All the optical transition energy data are collected from published papers for CNTs suspended in Sodium dodecyl sulfate (SDS) solutions. It is known that environmental effect can redshift the optical transition energy. Nugraha *et al.* [67] showed that the shift of optical transition energy due to environmental effect is related to dielectric constant of surrounding medium, nanotube diameter, subband index, and exciton size. To apply this algorithm for other surfactant, the energy shift relation [67] can be used for calculating *E_ii_* for any surfactant, hence extending the algorithm of chirality assignment for any type of nanotube environment. 

Table 3 shows the empirical equation based chirality assignment of 28 semiconducting SWCNTs using experimental values of *E*_11_ and *ω_rbm_* from reported RRS experiments [15,16,32,33,34,35,36,37,38,39,40,41,42,43]. From the table, 22 of the RRS data satisfy the conditions either 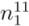 > 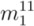 and 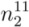 < 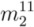 (mod 1) or 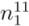 > 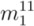
and 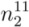 < 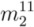 (mod 2), while two of them satisfy *round*(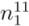) = *round*(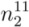) and *round*(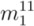) = *round*(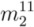 ) conditions. The remaining four semiconducting SWCNTs are further treated using error detection and refining method. For example, consider the actual chirality of (7, 3) whose (*n*, *m*) pairs are calculated as (6.7, 3.37) and (4.85, 5.4). Since these pairs satisfy the conditions 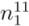 > 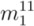 and 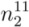 < 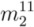 , the assigned mod type and the predicted chirality after rounding is 1 and (7, 3), respectively. The assigned mod type is the same as the predicted mod type (calculated from predicted chirality) in this case. Therefore, the mod type and chirality is 1 and (7, 3), respectively. Next, consider the actual chirality of (5, 4). The (*n*, *m*) pairs for mod 1 and mod 2 calculated from the empirical equations are (5.01, 3.99) and (4.83, 4.18), respectively. As rounding the values gives same pair, the assigned chirality is (5, 4). Lastly, consider the case where error detection and refining method is necessary. For the actual chirality of (13, 2), the (*n*, *m*) pairs are calculated as (8.17, 8.22) and (12.73, 2.57). These pairs satisfy the conditions 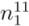 < 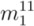 and 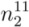 > 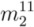, and consequently, the assigned mod type and the predicted chirality after rounding is 2 and (13, 3), respectively. Here, the assigned mod type is not the same as the predicted mod type (calculated from predicted chirality) in this case. The values that have fraction parts of *n* and *m* within 0.40 to 0.60, are prone to wrong assignment due to the error margin of our empirical equations or due to slight deviation in experimental data. Therefore, the valid pair is in the close vicinity of the actual chiral index. From the four possible nearest integer pairs, (12, 2), (12, 3), (13, 2), and (13,3), only (13,2) is mod 2 type. Therefore, (13, 2), which has the same mod type as the assigned mod type, is considered as the correct chiral index of that SWCNT. Following the presented algorithm, all 28 semiconducting SWCNTs were properly assigned with their actual chiral index from *E*_11_ and *ω_rbm_*. 

Table 4 shows the empirical equation based chirality assignment of 27 semiconducting SWCNTs using experimental values of *E*_22_ and *ω_rbm_* from reported RRS experiments [15,16,32,33,34,35,36,37,38,39,40,41,42,43]. From the table, 20 of the RRS data satisfy the conditions either 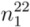 > 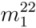 and 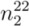 < 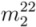 (mod 1) or 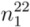 > 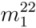 and 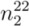 < 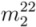 (mod 2), while four of them satisfy *round*(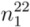) = *round*(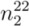) and *round*(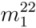 ) = *round*(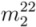) conditions. The remaining three semiconducting SWCNTs are further treated using error detection and refining method. Similar algorithm is applied to determine the actual chiral index from *E*_22_ and *ω_rbm_*. 

**Table 3:** Chirality Assignment of 28 semiconducting SWCNTs from *E*_11_ and *ω_rbm_*. Initially, 24 of them are rightly assigned as the predicted and assigned mod type matched. The remaining four semiconducting SWCNTs are further treated using error detection and refining method.

**RRS Data**		(*n*, *m*) pair **for mod 1**^*a*^		(*n*, *m*) pair **for mod 2**^*b*^		**Predicted chirality** ^*c*^	**Predi--cted** ^*d*^	Assi--gned^*e*^	**Re-assi--gned** ^*f*^	**Actual chiral**
*ω*_*rbm*_ (cm^−1^)	*E*_11_ (ev)	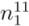	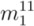	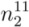	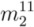	(*n*, *m*)	**Mod**	Mod	**Chirality**	(*n*, *m*)
373.0 [50]	1.488 [50]	5.01	3.99	4.83	4.18	(5, 4)	1	1		(5, 4)
335.2 [50]	1.420 [50]	5.28	4.79	6.03	3.97	(6, 4)	2	2		(6, 4)
329.5 [43]	1.249 [43]	6.7	3.37	4.85	5.4	(7, 3)	1	1		(7, 3)
309.0 [43]	1.283 [43]	6.04	4.9	5.97	4.98	(6, 5)	1	1		(6, 5)
304.0 [34]	1.362 [43]	5.5	5.65	8.84	1.47	(9, 1)	2	2		(9, 1)
297.0 [43]	1.306 [43]	5.78	5.64	7.78	3.36	(8, 3)	2	2		(8, 3)
291.0 [38]	1.100 [43]	8.75	2.32	5.28	6.37	(9, 2)	1	1		(9, 2)
283.0 [50]	1.212 [50]	6.39	5.62	7.01	4.95	(7, 5)	2	2		(7, 5)
280.0 [42]	1.110 [35]	7.85	4.11	5.79	6.36	(8, 4)	1	1		(8, 4)
263.0 [50]	1.117 [50]	6.49	6.48	9.75	2.54	(10, 3)	1	2	(10, 2)	(10, 2)
264.0 [43]	1.110 [43]	7.26	5.63	7.87	6.05	(7, 6)	1	1		(7, 6)
256.0 [37]	0.982 [50]	11.23	0.63	5.91	7.42	(11, 1)	1	1		(11, 1)
256.8 [43]	1.140 [43]	6.73	6.57	9.22	3.7	(9, 4)	2	2		(9, 4)
251.0 [50]	0.992 [50]	9.85	3.24	6.23	7.38	(10, 3)	1	1		(10, 3)
246.4 [39]	1.060 [35]	7.53	6.35	7.84	6.02	(8, 6)	2	2		(8, 6)
242.0 [42]	0.997 [50]	8.77	5.25	6.91	7.25	(9, 5)	1	1		(9, 5)
231.8 [42]	1.036 [50]	7.46	7.35	10.67	3.58	(11, 4)	1	2	(11, 3)	(11, 3)
229.0 [34]	0.979 [50]	8.41	6.57	8.04	6.96	(8, 7)	1	1		(8, 7)
226.0 [42]	0.901 [50]	11.89	2.29	6.82	8.37	(12, 2)	1	1		(12, 2)
221.8 [37]	0.904 [50]	10.74	4.34	7.19	8.32	(11, 4)	1	1		(11, 4)
215.0 [50]	0.937 [50]	8.63	7.41	9.25	6.73	(9, 7)	2	2		(9, 7)
213.4 [50]	0.898 [50]	9.81	6.24	8.01	8.17	(10, 6)	1	1		(10, 6)
210.9 [50]	0.949 [50]	8.17	8.22	12.73	2.57	(13, 3)	1	2	(13, 2)	(13, 2)
206.0 [34]	0.924 [50]	8.43	8.37	12.47	3.51	(12, 4)	2	2		(12, 4)
203.3 [39]	0.828 [50]	12.97	3.08	7.67	9.34	(13, 3)	1	1		(13, 3)
198.5 [39]	0.829 [50]	11.43	5.73	8.27	9.2	(11, 6)	2	1	(12, 5)	(12, 5)
192.5 [50]	0.841 [50]	9.78	8.25	10.27	7.73	(10, 8)	2	2		(10, 8)
187.2 [50]	0.835 [50]	9.49	9.12	12.82	5.26	(13, 5)	2	2		(13, 5)

^*a*^ Using Equations (8) and (10); ^*b*^ Using Equations (9) and (11); ^*c*^ After satisfying the conditions, *n* > *m* and *m*𝑜*d*(*n- m*, 3) ≠ 0; ^*d*^ Mod type is predicted from predicted chirality; ^*e*^ If the predicted chirality comes from (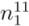, 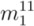) pair and (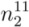, 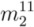) pair, the assigned mod type is mod 1 and mod 2, respectively; ^*f*^ If the predicted mod type and assigned mod type is not same, chiral index is selected from four index combination using error detection and refining method.

Although the algorithm is presented for chirality assignment of semiconducting SWCNTs, it can also detect the presence of any possible metallic CNTs. In practice, it is not easy to separate the metallic CNTs from semiconducting CNTs completely. Therefore, the detection of metallic CNTs using the algorithm is more helpful. For example, consider the metallic CNTs having chiral index of (8, 5), *ω_rbm_* of 262.7, and optical transition energy of 2.47 eV. Using the algorithm, two pairs (4.62, 8.20) and (3.96, 8.74) are calculated from Equations (8)-(11). Since both pairs follow the conditions, 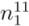 < 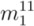 and 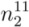 < 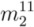, the sample is detected as metallic CNTs. 

**Table 4:** Chirality Assignment of 27 semiconducting SWCNTs from *E*_22_ and *ω*_*rbm*_. Initially, 24 of them are rightly assigned as the predicted and assigned mod type matched. The remaining four semiconducting SWCNTs are further treated using error detection and refining method.

**RRS Data**		(*n*, *m*) **pair for mod 1**^*a*^		(*n*, *m*) **pair for mod** 2^*b*^		**Predicted chirality** ^*c*^	**Predi--cted** ^*d*^	**Assi-** **-gned** **^*e*^**	**Re-assi--gned** ^*f*^	**Actual chiral**
*ω*_*rbm*_ (cm^−1^)	*E*_22_ (ev)	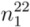	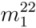	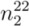	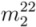	(*n*, *m*)	**Mod**	**Mod**	**Chirality**	(*n*, *m*)
309.0 [38]	2.180 [38]	6.01	4.94	5.44	5.52	(6, 5)	1	1		(6, 5)
304.0 [34]	1.800 [42]	5.07	6.07	9.22	0.83	(9, 1)	2	2		(9, 1)
299.0 [38]	1.860 [38]	5.33	6.03	7.71	3.35	(8, 3)	2	2		(8, 3)
283.0 [50]	1.920 [42]	6.01	6.01	6.69	5.34	(7, 5)	2	2		(7, 5)
278.8 [43]	2.110 [42]	7.62	4.45	5.69	6.51	(8, 4)	1	1		(8, 4)
264.6 [39]	1.690 [39]	5.93	6.95	9.98	2.09	(10, 2)	2	2		(10, 2)
264.2 [37]	1.910 [37]	6.98	5.92	6.54	6.38	(7, 6)	1	1		(7, 6)
257.5 [39]	1.720 [39]	6.31	6.95	8.55	4.51	(9, 5)	1	2	(9, 4)	(9, 4)
245.0 [42]	1.720 [42]	6.98	6.99	7.92	6.02	(8, 6)	2	2		(8, 6)
242.0 [42]	1.850 [42]	8.66	5.37	6.69	7.46	(9, 5)	1	1		(9, 5)
236.0 [50]	1.556 [50]	6.65	7.88	12.24	0.69	(12, 1)	2	2		(12, 1)
233.0 [50]	1.565 [50]	6.84	7.89	11.04	2.95	(11, 3)	2	2		(11, 3)
230.0 [42]	1.700 [42]	8.08	6.85	7.63	7.31	(8, 7)	1	1		(8, 7)
227.0 [50]	1.820 [37]	11.88	2.21	6.76	8.37	(12, 2)	1	1		(12, 2)
226.0 [46]	1.570 [46]	7.29	7.93	9.77	5.22	(10, 5)	2	2		(10, 5)
221.8 [37]	1.760 [37]	11.16	3.77	7.04	8.47	(11, 4)	1	1		(11, 4)
216.0 [39]	1.564 [39]	8.20	7.74	8.68	7.25	(9, 7)	2	2		(9, 7)
212.0 [42]	1.640 [50]	9.98	6.16	7.74	8.55	(10, 6)	1	1		(10, 6)
207.1 [46]	1.447 [46]	7.85	8.85	11.9	4.21	(12, 4)	2	2		(12, 4)
204.0 [37]	1.535 [37]	9.36	7.65	8.64	8.39	(9, 8)	1	1		(9, 8)
203.0 [42]	1.620 [42]	12.49	3.83	7.73	9.31	(12, 4)	2	1	(13, 3)	(1, 3)
200.0 [34]	1.440 [34]	8.45	8.88	10.66	6.51	(11, 7)	1	2	(11, 6)	(11, 6)
197.7 [46]	1.560 [46]	11.73	5.44	8.12	9.42	(12, 5)	1	1		(12, 5)
192.5 [50]	1.428 [50]	9.28	8.78	9.94	8.08	(10, 8)	2	2		(10, 8)
189.3 [46]	1.479 [46]	11.42	6.77	8.72	9.66	(11, 7)	1	1		(11, 7)
183.0 [34]	1.466 [34]	14.14	4.00	8.63	10.40	(14, 4)	1	1		(14, 4)
183.3 [50]	1.390 [50]	10.27	8.74	9.88	9.15	(10, 9)	1	1		(10, 9)

^*a*^ Using Equations (12) and (14); ^*b*^ Using Equations (13) and (15); ^*c*^ After satisfying the conditions, *n* > *m* and *m*𝑜*d* (*n- m*, 3) ≠ 0; ^*d*^ Mod type is predicted from predicted chirality; ^*e*^ If the predicted chirality comes from (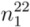, 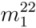) pair and (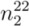, 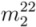) pair, the assigned mod type is mod 1 and mod 2, respectively; ^*f*^ If the predicted mod type and assigned mod type is not same, chiral index is selected from four index combination using error detection and refining method.

## 5. Conclusions

The empirical equation based chirality assignment presents a novel technique of assigning SWCNT chirality by solving a set of empirical equations. A set of effective empirical equations for tight binding model hopping parameter is proposed to predict first and second optical transition energies (*E*_11_ and *E*_22_). All the empirical equations contain a term (2*n*− *m*) to reﬂect the “chirality effect”. Using values of RBM frequency and any one of the first or second optical transition energies (*E*_11_ or *E*_22_) from RRS, the empirical equations for the (2*n*− *m*) term are solved to provide the chiral index of the unknown semiconducting SWCNT. In total, 28 semiconducting SWCNTs were assigned using *E*_11_ and *ω_rbm_* values and another 27 semiconducting SWCNT were assigned using *E*_22_ and *ω_rbm_* values. Moreover, the procedure for the detection of metallic CNTs using the algorithm is also presented. 

Unlike existing methods of chirality assignment, this technique does not require graphical comparison or pattern recognition between existing experimental plot and theoretical plot. The technique is especially useful for determining chirality of isolated nanotube that does not get the advantage of pattern recognition from a produced batch of SWCNTs. This technique of chirality assignment also validates the empirical equations of band gap energy, *E*_11_ and *E*_22_ that were used to assign chirality by accessing them from reverse direction. Though the chirality assignments of some of the samples were detected and refined for the erroneous cases, there remains the possibility of obtaining truly ambiguous results from this method in some cases. In fact, so far no single proposed method for chirality assignment can be independent, and this technique may also generate some ambiguous cases that require verification by other methods. Therefore, our proposed technique for chirality assignment should also be taken under that perspective. In the future, further attempts may be taken to make this method more effective as well as to extend it to the chirality assignment of metallic CNTs. 

## Appendix

### A. Chirality Assignment from First Optical Transition Energy

The relation of RRS given *ω_rbm_* with SWCNT diameter (d*_t_*) is established by semi-empirical equation, *ω_rbm_* = 223.5/*d_t_* + 12.5, as proposed by many authors [16,33,34,50]. This relation gives

(16)

For a*_cc_* = 0.144 nm, using the known expression of  , we get,

(17)

As it is not known at this point whether the sample under test is mod 1 or mod 2 type, both Equations (4) and (5) are considered to find the value of 2*n*− *m* in terms of *d_t_* and *E*_11_. Equations (2) and (4) give,

(18)

and Equations (2) and (5) give,

(19)

Using the value of *ω_rbm_* and *E*_11_ from RRS experiment, the right hand sides of Equations (17)-(19) can be known. The values of *n* from the first optical transition energy for mod 1 and mod 2 is termed as  and , respectively. Solving Equations (17) and (18) for  gives

(20)

Similarly, solving Equations (17) and (19) for  gives

(21)

Each of the above two expressions of  and  gives two values for (+) and (−) terms. The values of  and  corresponding to the (−) terms of the above two expressions were always found to give negative value and hence become invalid as the chirality index (*n* and *m*) cannot be negative. So, only the values of  and  corresponding to the (+) terms can be taken for consideration.

From Equations (18) and (19), the corresponding values of  and  are  = 2 - *a*_1_ and  = 2 - a2, respectively. As the values of  and  and the corresponding  and  may become fractions, they are rounded to nearest integers. These give two integer pairs (, ) and (, ) who are the candidates for chiral index of unknown SWCNT.

### B. Chirality assignment from Second Optical Transition Energy

Just like before, as it is not known at this point whether the sample under test is mod 1 or mod 2, both Equations (16) and (17) are considered to find the value of 2*n*− *m* in terms of *d_t_* and *E*_22_. Equations (3) and (6) yield

(22)

and Equations (3) and (7) give

(23)

Using the value of *ω_rbm_* and *E*_22_ from RRS experiment, the right hand sides of Equations (17), (22), and (23) can be known. The values of *n* from the second optical transition energy for mod 1 and mod 2 is termed as  and , respectively. Solving Equations (17) and (22) for  gives

(24)

Similarly, solving Equations (17) and (23) for  gives

(25)

Again, each of the above two expressions of  and  gives two values for the (+) and (−) terms. Just like before, the values of  and  corresponding to the (−) terms of the above two expressions were always found to give negative value and become invalid. Only the values of  and  corresponding to the (+) terms can be taken.

From Equations (22) and (23), the corresponding values of  and  are  = 2 - *b*_1 _and  = 2 - *b*_2_, respectively. As the values of  and  and the corresponding  and  may become fractions, they are rounded to nearest integers. These give two integer pairs ( ,  ) and (,  ) who are the candidates for chiral index of unknown SWCNT.
